# Estimation of heat transfer coefficient and friction factor with showering of aluminum nitride and alumina water based hybrid nanofluid in a tube with twisted tape insert

**DOI:** 10.1038/s41598-023-49142-w

**Published:** 2023-12-27

**Authors:** Wathek Chammam, Umar Farooq, Mirwais Sediqmal, Hassan Waqas, Sumeira Yasmin, Fakhar Zulfiqar, Dong Liu, Shan Ali Khan

**Affiliations:** 1 Department of Mathematics, College of Science, Majmaah University, 11952 Al-Majmaah, Saudi Arabia; 2 Department of Mechanical and Computer-Aided Engineering, National Formosa University, Yunlin, Taiwan, R. O. C.; 3 Civil Engineering Department, Engineering Faculty, Laghman University, Mehtarlam, Laghman 2701 Afghanistan; 4 School of Energy and Power Engineering, Jiangsu University, Zhenjiang, 212013 China; 5 Department of Mathematics, Government College University Faisalabad, Faisalabad, 38000 Pakistan

**Keywords:** Energy science and technology, Engineering, Mathematics and computing, Nanoscience and technology

## Abstract

Twisted tape is one of the active thermal proficiency boosting technology which has been deeply examined because to consistent efficiency findings and easy implementations. Thermo-hydraulic effectiveness of tubes fitted with twisted tapes is becoming highly significant. Although twisted tapes can cause swirls and disturb boundary layers, this is the most widely used method for improving convection. In the present attempt, to enhance the heat transfer twisted tape is inserted in tube. In the current modern research, the effect of twisted tape, on the enhancement of thermal transport, Nusselt number and friction factor performance of *AIN*–Al_2_O_3_/water hybrid nanofluid is evaluating utilizing CFD and ANSYS-FLUENT software. the consequence of twisted pitch 44 mm, 66 mm, 88 mm, 100 mm and Reynolds numbers 800, 1200, 1600 and 2000 on Nusselt number, heat transfer coefficient and friction coefficient have been computed numerically with 0.01 to 0.04 volume friction of nanopowders. The commercial ANSYS-FLUENT code was used in this analysis utilizing the SIMPLE method for pressure–velocity coupling. The $K - \omega$ model and Navier Stokes equations are integrating utilizing finite volume method in ANSYS-FLUENT. It was observed that inserting the twisted tape in tube significantly improves the thermal conductivity as well as friction factor compared with the simple tube without turbulator.

## Introduction

Energy demand has increased significantly in recent years around the world as a result of rising population and improved transportation and technology. Nanotechnology is one of the latest passive strategies employed by various researchers. Various nanopowders can be utilized for solar implementations, and among them, Aluminum oxide has piqued researchers' interest. The majority of required energy is produced by nonrenewable or fossil fuels, which are in short supply due to global demand^1^. Al-Kayiem et al.^2^ discussed the applications of twisted tape in nanotechnology. Contaminations caused by burning these sorts of fuels have negative consequences for the environment, including climate change and, as a result, worldwide warming. As a result, the form of energy used should be changed to one that is more ecologically friendly. The greatest option for achieving this goal is to use renewable energy sources^3^. Solar energy can fulfill the world's energy demand because it is limitless and free. Fattahi^4^ discussed the hybrid nanofluid flow for numerical computations of solar collector and twisted tape inserted. Behura et al.^5^ analyzed the heat efficiency of solar collector with the help of twisted tape.

The use of swirl flow generators is one of the most attractive passive strategies that has recently become crucial to enhance the rate of thermal efficiency and boost the performance of thermal devices for several implementations. Swirl flow generators, which provide excellent flow mixing at a low cost, are practical and cost-effective heat transfer innovation technology. Twisted tapes are one of the foremost often employed swirl generators for improving heat transfer characteristics, particularly in heating systems^6^.The twisted tapes come in a variety of shapes and sizes, and they can disrupt the thermal barrier layer by generating severe swirl flows that cause intense flow mixing and, as a result, greater heat transfer coefficients^7^. Results indicate that the highest thermal efficiency factors for PATT, PTT, and TT, accordingly, are 1.433, 1.396, and 1.24 at constant pumping power. Wang et al.^8^ analyzed the solar collector performance in the presence of nanopowders and twisted turbulator. The behavior of heat and fluid flow in a laminar flow through tube with twisted tape inserted were examined by Manjunath^9^. Sheikholeslami et al.^10^ analyzed the capability of solar heat exchanger with twisted turbulator. They concluded that the heat transfer is enhanced by changing the nanoparticles shape spherical to blade form.

The normal working fluids possess low heat proficiency and therefore, replacing them with the fluids with greater heat efficiencies can be an effective approach to boost the rate of heat transfer. The invention of nanofluids marked a turning point in heat transfer technology. Nanofluids are liquids in which solid nanomaterials are dispersed. Another way to minimize energy waste and advance the overall performance of thermal devices is to use nanofluids as base fluid in a range of manufacturing and engineering applications.

Numerous ways for improving heat transfer in various systems, such as microchannel, have been created in recent years, and many researchers have employed these approaches to enhance their productivity and effectiveness. Nanofluids may improve heat transmission due to their better thermal characteristics to base fluids, according to open literature. Noorbakhsh et al.^11^ examined the nanofluid flow through double pipes with twisted tape inserted. Algarni^12^ investigate the hybrid nanofluid flow through helical twisted tape insert in tube. Farshad and Sheikholeslami^13^ analyzed the behavior of exergy and entropy of system in nanofluid flow inside solar collector with twisted tape. Alnaqi et al.^14^ disclosed the ${\text{MgO}} - MWCNTs$/thermal oil hybrid nanofluid flow with inserted twisted tube in solar collector. Sheikholeslami and Jafaryar^15^ investigated the thermal performance by using CNTS with helical tabulator. The effect of solar system with using hybrid nanoparticles was investigated by Sheikholeslami ^16^.

The novelty of this analysis is to compute the thermal transport, Nusselt number and friction factor performance of $AIN - {\text{Al}}_{2} {\text{O}}_{3} /{\text{water}}$ hybrid nanofluid subjected to twisted tape inserted in tube. The laminar flow and heat transfer is scrutinized. ANSYS-FLUENT software is used to compute the results of current developed model. Potential motivations for using hybrid nanofluids include enhancing heat transfer efficiency, improving energy efficiency, and optimizing nanofluid parameters. Research gaps may involve investigating the specific applications and industries where enhanced heat transfer is critical, comparing the performance of different nanoparticles types and concentrations, addressing environmental and safety concerns, and conducting cost–benefit analyses to assess economic feasibility. These considerations could provide a basis for emphasizing the advantages of hybrid nanofluids and addressing knowledge gaps in their practical application and optimization.

## Geometric model and formulation

**Figure 1:**
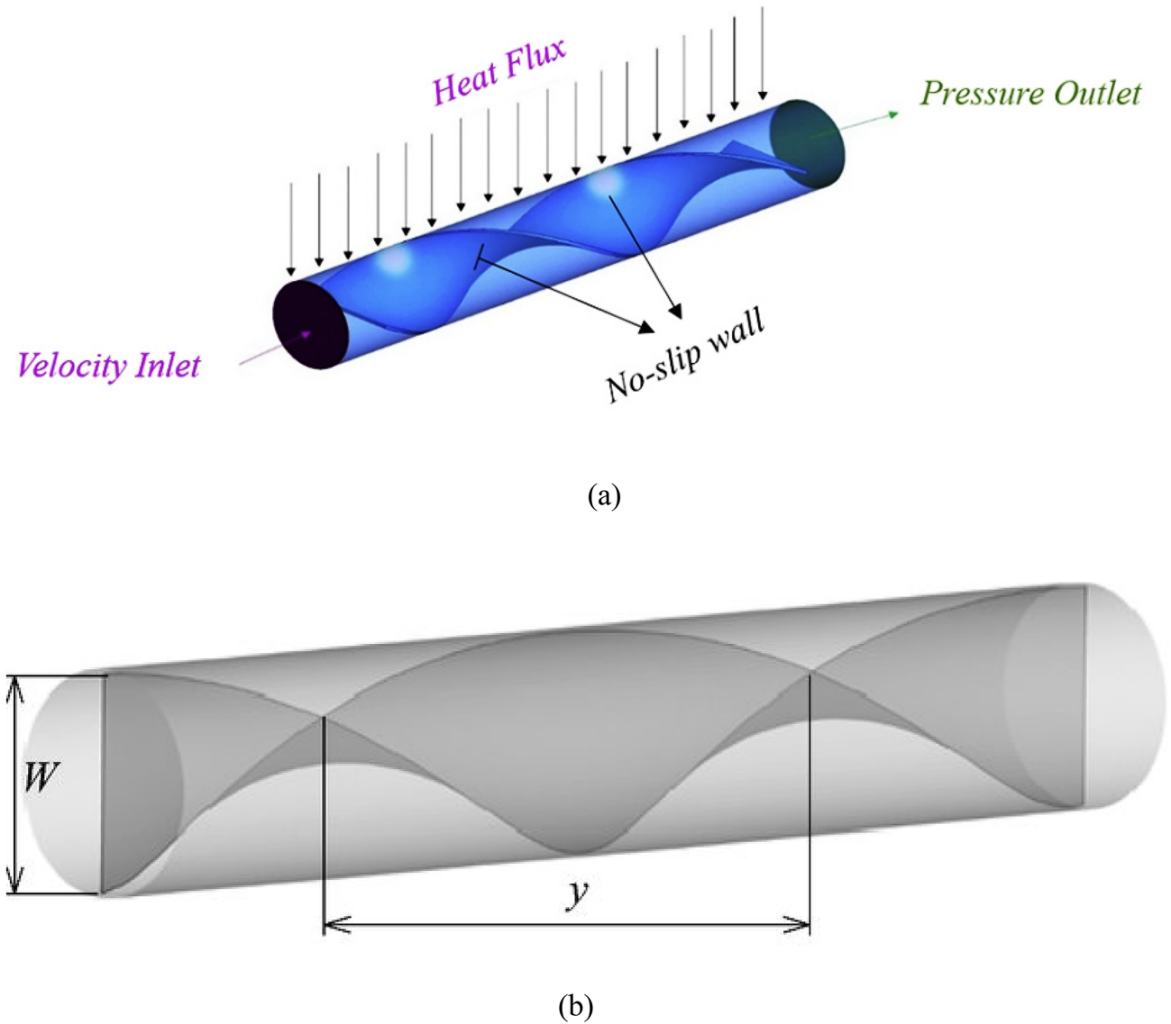
(**a**) Problem schematic. (**b**) Tube with twisted tape.

Here, the twisted tape inserted in tube for hybrid nanofluid containing an alumina nitride $AIN$ and alumina oxide ${\text{Al}}_{2} {\text{O}}_{3}$ nanopowders with 30 nm diameter size is considered. The twisted tape is inserted in full length of tube. In this analysis, the 3D, $AIN - {\text{Al}}_{2} {\text{O}}_{3} /{\text{water}}$ hybrid nanofluid flow with steady state condition problem is formulated. In the current advanced research the range of Reynolds number 800, 1200, 1600 and 2000 considered. Figure 1 indicates the flow geometry tube with twisted tape. The dimensions of tube and twisted tape are listed in Table 1.

**Table 1:** Dimensions of tube and pitch of twisted tape for 180° rotation.

Tube type	Thickness (t) (mm)	Diameter (d) (mm)	Length (L) (mm)	Twist pitch (p)
Simple tube	0	22	2200	0
PR = 2	1	22	2200	44 mm
PR = 3	1	22	2200	66 mm
PR = 4	1	22	2200	88 mm
PR = 5	1	22	2200	110 mm

The governing system is presented as follows^17^:

- Continuity equation:
  1 $$\partial_{{x_{i} }} \rho_{eff} u_{i} = 0,$$
- Momentum equation:
  2 $$\begin{gathered} \rho_{eff} \partial_{{x_{i} }} \left( {u_{i} u_{j} } \right) = - \partial_{{x_{i} }} p + \partial_{{x_{j} }} \tau_{ij} + \rho_{eff} g_{i} , \hfill \\ \tau_{ij} = \mu^{**} \partial_{{x_{j} }} u_{i} \hfill \\ \mu^{**} = \mu_{eff} + \mu_{t} \hfill \\ \end{gathered}$$
- Energy equation:
  3 $$\begin{gathered} \partial_{{x_{i} }} \left( {\rho_{eff} c_{p,eff} u_{i} T} \right) = \partial_{{x_{i} }} \left( {\lambda^{**} \partial_{{x_{i} }} T} \right), \hfill \\ \lambda^{**} = \lambda + \lambda_{t} \hfill \\ \lambda_{t} = \frac{{c_{p,eff} \mu_{t} }}{{\sigma_{t} }}. \hfill \\ \end{gathered}$$

In this analysis the $k - \omega$ turbulence model is implemented. This model has ability to covers the weaknesses of the model. This model is used due to larger accuracy and convergence speed. The following turbulent formulations are used:

4 $$\partial_{{x_{i} }} \left( {\rho_{eff} u_{i} k} \right) = \partial_{{x_{i} }} \left[ {\left( {\mu_{eff} + \frac{{\mu_{t} }}{{\sigma_{k} }}} \right)\partial_{{x_{i} }} k} \right] + G^{*}_{k} - Y^{*}_{k} ,$$

5 $$\partial_{{x_{i} }} \left( {\rho_{eff} u_{i} \omega } \right) = \partial_{{x_{i} }} \left[ {\left( {\mu_{eff} + \frac{{\mu_{t} }}{{\sigma_{\omega } }}} \right)\partial_{{x_{i} }} k} \right] + G^{*}_{\omega } - Y^{*}_{\omega } ,$$

6 $$\begin{gathered} \mu_{t} = \frac{{\rho_{eff} k}}{\omega }{1 \mathord{\left/ {\vphantom {1 {\max \left\{ {{1 \mathord{\left/ {\vphantom {1 {\alpha^{**} ,{{\Omega F_{2} } \mathord{\left/ {\vphantom {{\Omega F_{2} } {a_{1} \omega }}} \right. \kern-0pt} {a_{1} \omega }}}}} \right. \kern-0pt} {\alpha^{**} ,{{\Omega F_{2} } \mathord{\left/ {\vphantom {{\Omega F_{2} } {a_{1} \omega }}} \right. \kern-0pt} {a_{1} \omega }}}}} \right\}}}} \right. \kern-0pt} {\max \left\{ {{1 \mathord{\left/ {\vphantom {1 {\alpha^{**} ,{{\Omega F_{2} } \mathord{\left/ {\vphantom {{\Omega F_{2} } {a_{1} \omega }}} \right. \kern-0pt} {a_{1} \omega }}}}} \right. \kern-0pt} {\alpha^{**} ,{{\Omega F_{2} } \mathord{\left/ {\vphantom {{\Omega F_{2} } {a_{1} \omega }}} \right. \kern-0pt} {a_{1} \omega }}}}} \right\}}} \hfill \\ \Omega = \sqrt {2\Omega_{ij} \Omega_{ij} } \hfill \\ \end{gathered}$$

The rotation tensor mean rate is addressed as:

7 $$\Omega_{ij} = 0.5\left( {\partial_{j} u_{i} - \partial_{i} u_{j} } \right)$$

The temperature at the tube wall is constant. The pressure-outlet constraint is employed at outlet face. There is no slip on the tube and twisted tape wall.

### Boundary conditions

The boundary conditions of this problem are as follows:

- Heat flux distribution along outer wall of the tube is uniform whereas the wall of inner inserted twisted tape is adiabatic.
- In the present study, conduction heat transfer in the tube and the tape has been taken into account.
- In momentum equation, no-slip boundary condition is used for all the walls of the receiver.
- The velocity inlet and pressure outlet are set in inlet and outlet of the tube, respectively.

### Hybrid nanofluid characteristics

The hybrid combination of nitride and aluminum oxide nanoparticles dispersed in base fluid as water. The Thermophysical characteristics such as density, thermal conductivity, specific heat and viscosity of hybrid nanofluid have been deliberated. To predict the nature of hybrid nanofluid, the following modeling has been used:

8 $$\rho_{eff} = \left( {0.01\phi } \right)\phi_{p} + \left( {1 - 0.01\phi } \right)\rho_{f} ,$$

9 $$\frac{{k_{eff} }}{{k_{f} }} = 1.2035\left[ {\left( \begin{gathered} 0.001 \hfill \\ + 0.01\phi \hfill \\ \end{gathered} \right)^{0.0098} \left( \begin{gathered} 0.01 \hfill \\ + {{T_{nf} } \mathord{\left/ {\vphantom {{T_{nf} } {90}}} \right. \kern-0pt} {90}} \hfill \\ \end{gathered} \right)^{0.1331} \left( \begin{gathered} 0.001 \hfill \\ + {{dp} \mathord{\left/ {\vphantom {{dp} {170}}} \right. \kern-0pt} {170}} \hfill \\ \end{gathered} \right)^{ - 0.0001} \left( \begin{gathered} 0.01 \hfill \\ + {{\alpha_{p} } \mathord{\left/ {\vphantom {{\alpha_{p} } {\alpha_{f} }}} \right. \kern-0pt} {\alpha_{f} }} \hfill \\ \end{gathered} \right)^{0.0153} } \right],$$

10 $$C_{eff} = \frac{{\left( {0.01\phi } \right)\left( {\rho C} \right)_{p} + \left( {1 - 0.01\phi } \right)\left( {\rho C} \right)_{f} }}{{\rho_{eff} }},$$

11 $$\frac{{\mu_{eff} }}{{\mu_{f} }} = 0.3659 \times C1 \times exp\left[ {\left( {1 + 0.01\phi } \right)^{10.83} \left( {{{T_{nf} } \mathord{\left/ {\vphantom {{T_{nf} } {90}}} \right. \kern-0pt} {90}}} \right)^{ - 0.0239} \left( {1 + {{dp} \mathord{\left/ {\vphantom {{dp} {170}}} \right. \kern-0pt} {170}}} \right)^{ - 0.1609} } \right].$$

Here $\phi$ denotes the volume fraction of hybrid nanofluid, the subscript symbols such as $f$ designates the fluid, $p$ be the solid nanopowders and $eff$ for hybrid nanofluid. The formulated values of Thermophysical properties of hybrid nanofluid from Eqs. (4–7) can be listed in Table 2. The Thermophysical values of particles and base fluid are listed in Table 3.

**Table 2:** Calculated values of Thermophysical properties by using expressions (4–7).

Volume concentration	Density $\left( {{\text{kg}}/{\text{m}}^{3} } \right)$	Specific heat $\left( {J/kg\;K} \right)$	Thermal conductivity $\left( {W/{\text{m}}\;{\text{K}}} \right)$	viscosity $\left( {{\text{kg}}/{\text{m}}\;{\text{s}}} \right)$
0.01	1105	4149	0.652	0.0082
0.02	1126	4115	0.765	0.0087
0.03	1145	4095	0.814	0.0092
0.04	1157	4083	0.895	0.0095

**Table 3:** Thermophysical characteristics of involved materials (base fluid & nanoparticles)^18^.

Property	${\text{H}}_{2} {\text{O}}$	${\text{AIN}}$	${\text{Al}}_{2} {\text{O}}_{3}$
$\rho \left( {{\text{kg}}\;{\text{m}}^{ - 3} } \right)$	998	3260	3880
$C_{p} \left( {{\text{J}}\;{\text{kg}}^{ - 1} \;{\text{K}}^{ - 1} } \right)$	4180	735	773
$k\left( {W\;m^{ - 1} \;{\text{K}}^{ - 1} } \right)$	0.6067	180	40
$\mu \left( {{\text{kg}}^{ - 1} \;{\text{m}}^{ - 1} \;{\text{s}}^{ - 1} } \right)$	0.0014	–	–

The dimensionless parameters are used in this communication:

### Reynolds number

The Reynolds number can be addressed as^17^

12 $$Re = \frac{{\rho_{eff} vD}}{\mu }.$$

    Here $D$ denotes the internal diameter of tube.

### Nusselt number

The heat transfer coefficient is evaluating by^19^:

13 $$h_{eff} = Q/A_{s} \left( {T_{s} - T_{eff} } \right).$$

    Here

The heat transfer rate is calculated by the following expression:

14 $$Q = m_{eff} C_{peff} \left( {T_{out} - T_{in} } \right)_{eff} .$$

where $m_{eff}$ denotes the mass flow rate, $T_{out}$ is the temperature and $T_{in}$ indicates inlet temperature.

Here $A_{s}$ represents the heat transfer area, $T_{s}$ indicates wall average temperature and $T_{eff}$ signifies the average temperature of hybrid nanofluid.

The Nusselt number is addressed as^17^

15 $$Nu = \frac{hD}{{k_{eff} }}.$$

where $h$ the convective heat is transfer coefficient and $k_{eff}$ denotes thermal conductivity.

### Friction factor

The friction factor can be written as^20^:

16 $$f_{r} = \frac{2\Delta p\,}{{\rho_{eff} v^{2} }}\frac{D}{L}.$$

Here $\Delta p$ is the pressure difference and $L$ be the length of tube.

## Numerical modeling

ANSYS FLUENT software (version 19.0) is used to compute the simulation of current model. The governing equations and $k - \omega$ turbulence model is numerically computed through the finite volume method. The convective and diffusive terms are resolve by utilizing second order scheme. Furthermore, the SIMPLE strategy is employed to compute the pressure–velocity coupling. The convergence criteria are set to $10^{ - 6}$ for all governing equations. In the selected model, *Y*^+^ for all simulations should be less than 5. Y^+^ enhance with increasing inlet velocity while the highest value is 3.377 and satisfies the above restriction.

## Results validation

From Table 4 the good agreement is observed between experimental analysis and current study for Nusselt number on different values of Reynolds number.

**Table 4:** Results validation with experimental study and current study for Nusselt number.

Reynolds Numbers	Experimental	Numerical	For PR = 5
	Manglik and Bergles^21^	Present study	
	Nusselt Number	Nusselt Number	Error %
800	24.69026549	22.6494	8.265870968
1200	33.71681416	34.0698	1.046913386
1600	41.81415929	39.5733	5.359092063
2000	49.77876106	44.437	10.73100444

### Grid independence test

**Figure 2:**
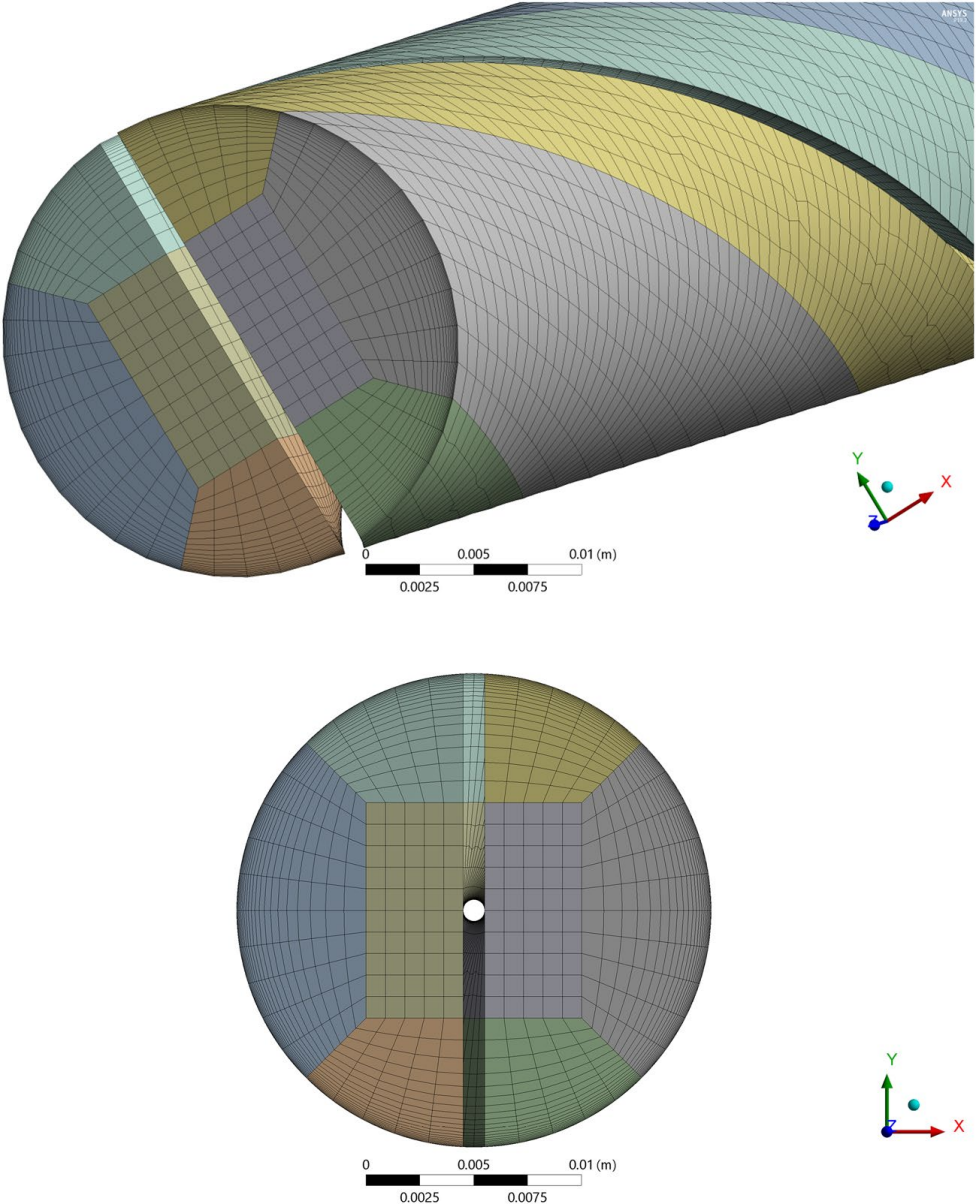
The computational mesh of current simulation.

In order to analyze the grid independence, the heat transfer coefficient and Nusselt number for Aluminum Nitride and Alumina/water based hybrid nanofluid in tube with twist pitch 44 mm is obtained for different grid point. From the simulation it is concluded that elements 96,800 is more sufficient for the computations because at this grid high heat transfer and Nusselt number obtained as shown in Table 5. Figure 2 examines developed grid meshes in current analysis for tube with twisted tape inserts.

**Table 5:** Meshes considered for analysis.

Mesh type	Nodes	Elements	HTC	NN
Coarse	76,992	64,000	972.645	35.6637
Normal	112,280	96,800	1201.69	44.0619
Fine	140,216	124,904	1178.45	39.4228

## Result and discussion

In this portion, the outcomes of the current numerical simulation are displayed. The impact of different pitch ratio of twisted tape on Nusselt number, heat transfer coefficient, and friction factor is scrutinized. Moreover the contours plots of velocity, temperature, and pressure are presented for different cases (a) simple tube (b) PR = 2 (c) PR = 3 (d) PR = 4 (e) PR = 5 and $\phi = 4\%$ with $Re = 2000$.

**Figure 3:**
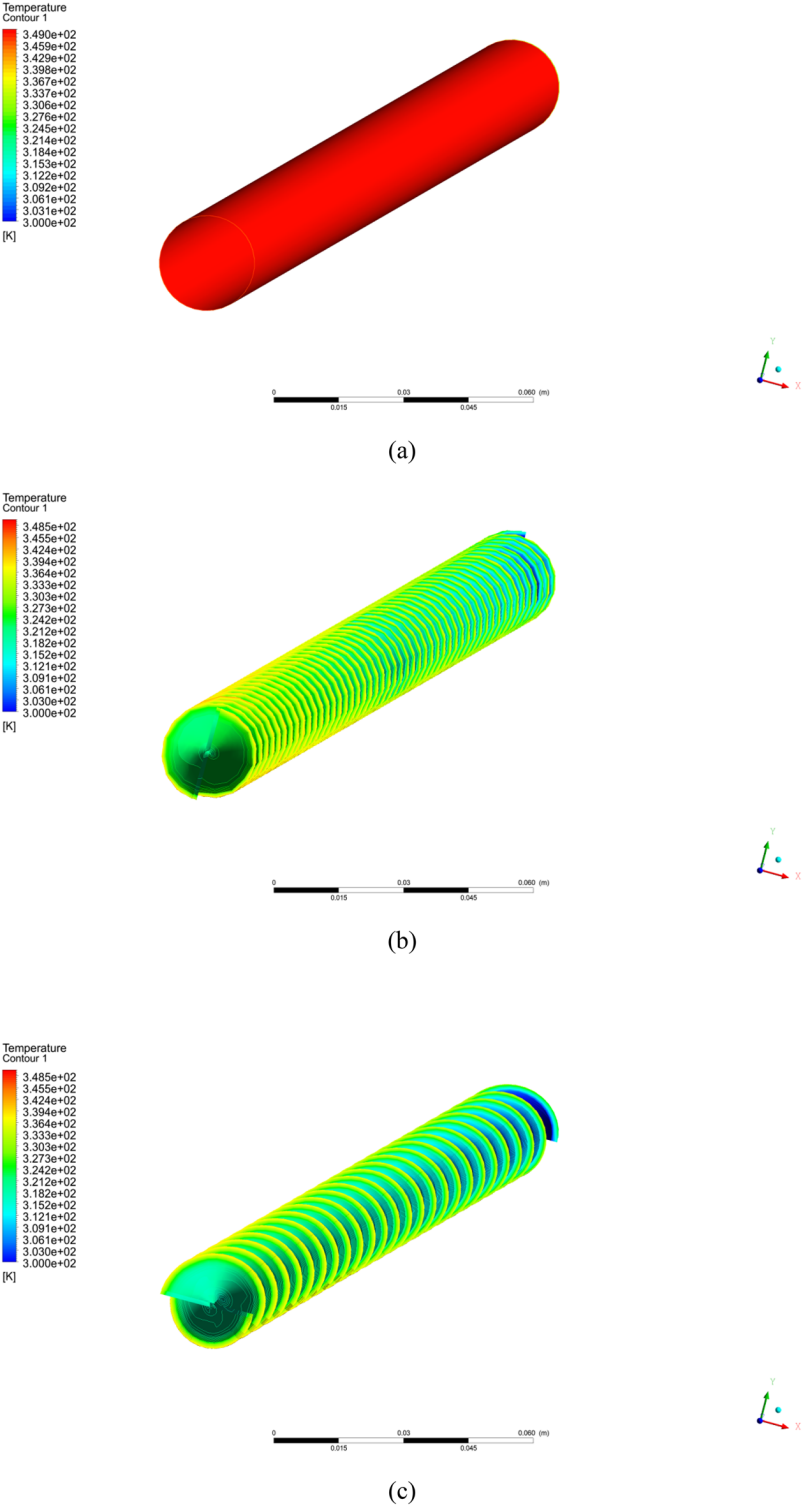

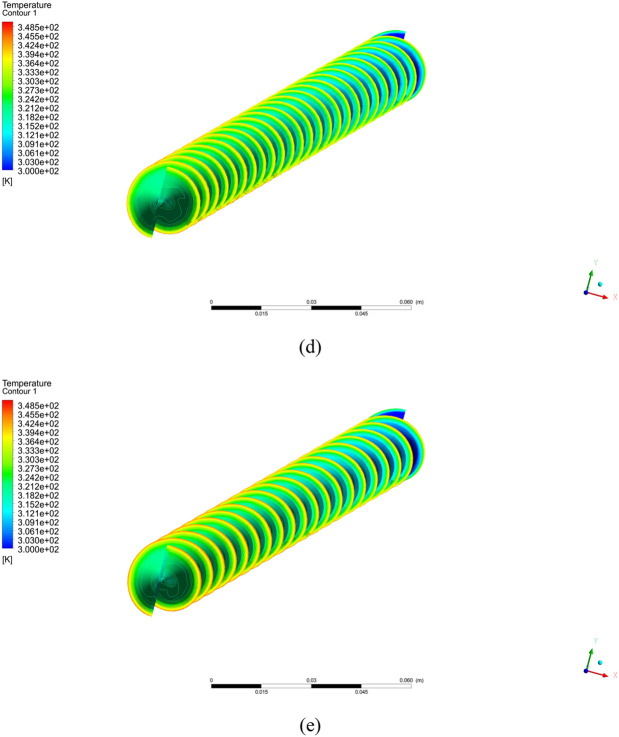
Temperature contour for hybrid nanofluid with $Re = 2000$ and $\phi = 4\%$ for the captured cases (**a**) simple tube (**b**) PR = 2 (**c**) PR = 3 (**d**) PR = 4 (**e**) PR = 5.

**Figure 4:**
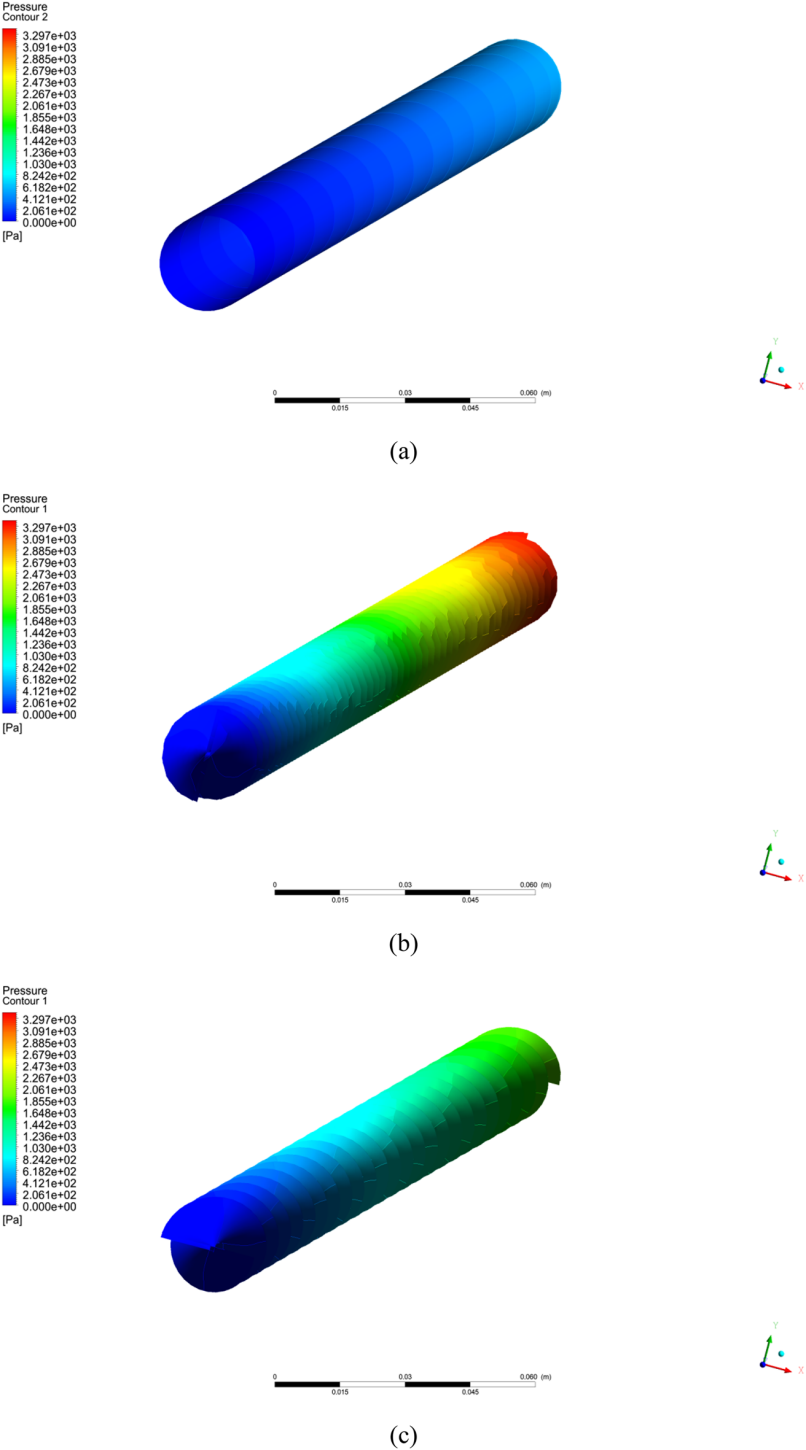

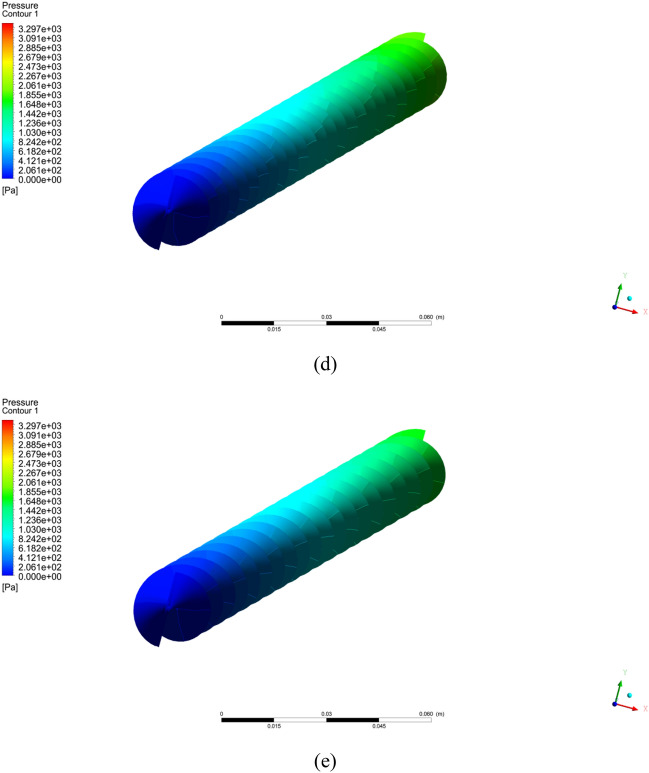
Pressure contour for hybrid nanofluid with $Re = 2000$ and $\phi = 4\%$ for the captured cases (**a**) simple tube (**b**) PR = 2 (**c**) PR = 3 (**d**) PR = 4 (**e**) PR = 5.

Figure 3 shows the temperature contours for simple tube and twisted tape inserted in tube with pitch ratio PR = 2 (c) PR = 3 (d) PR = 4 (e) PR = 5 at Reynolds number is 2000 and $\phi = 4\%$. As can be noticed that temperature is increases with reduce the twist ratio and increment in Reynolds number. The pressure contours are display in Fig. 4 for hybrid nanofluid with volume friction is $0.04$ and higher $Re = 2000$ for simple tube and inserted twist tape in tube with pitch ratio (b) PR = 2 (c) PR = 3 (d) PR = 4 (e) PR = 5. It can be analyzed that the pressure is increased with reducing the pitch ratio of twisted tape. In Fig. 8 the turbulence kinetic energy contours are elaborated.

### Impact of pitch ratio on Nusselt number

**Figure 5:**
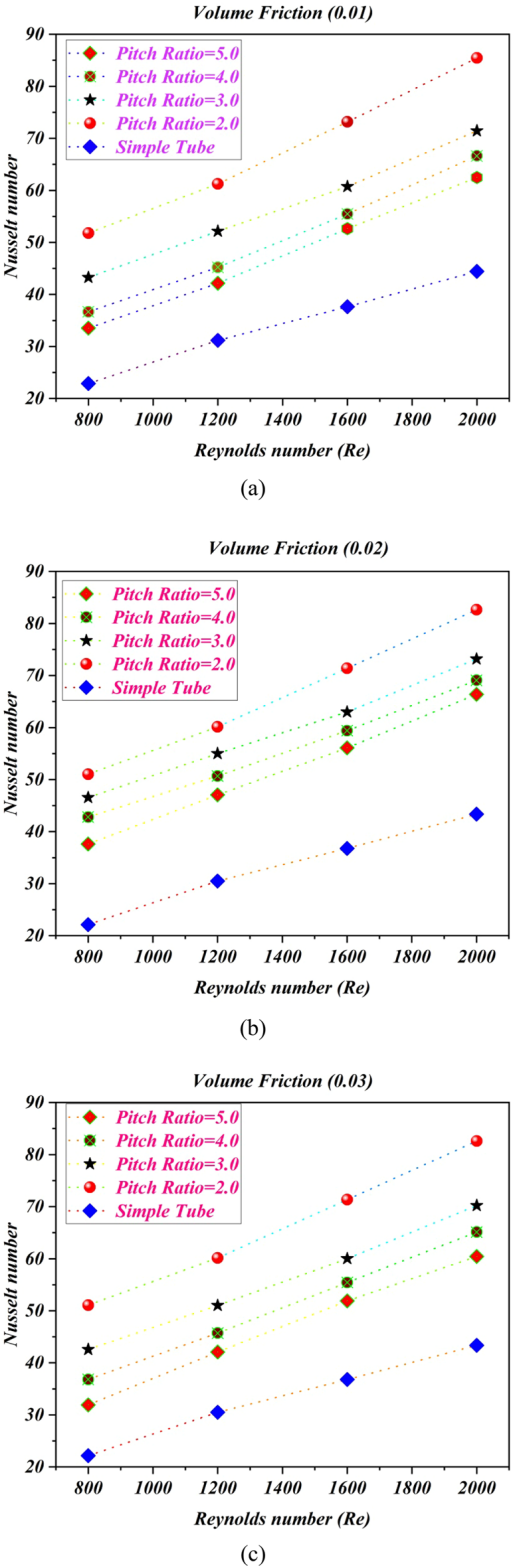

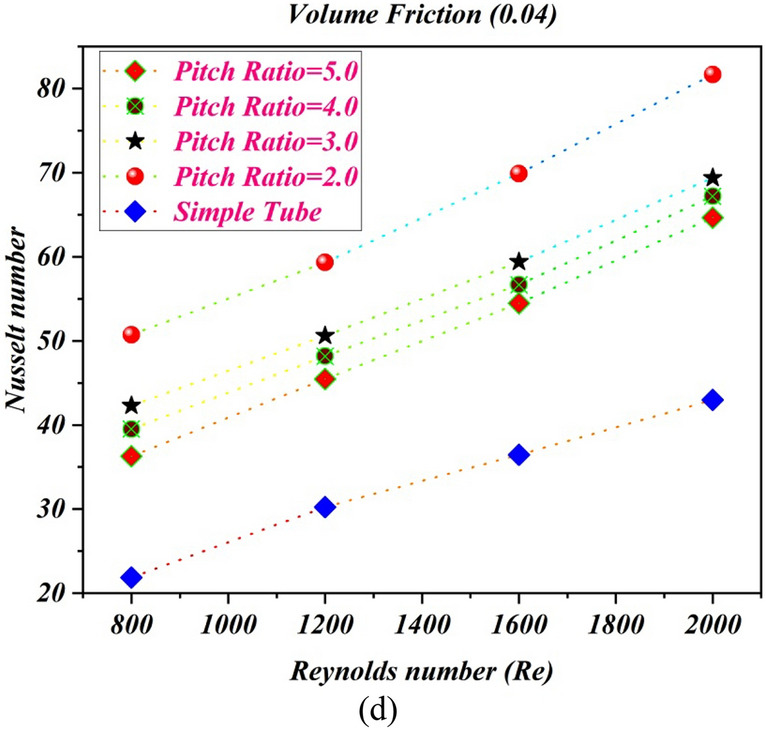
The estimations of Nusselt number against $Re$ with captured different cases for (**a**) $\phi = 1\%$ (**b**) $\phi = 2\%$ (**c**) $\phi = 3\%$ (**d**) $\phi = 4\%$.

Figure 5 shows the estimations of Nusselt number against Reynolds number with different pitch ratio and simple tube for various volume fractions of nanopowders (a) $\phi = 1\%$ (b) $\phi = 2\%$ (c) $\phi = 3\%$ and (d)$\phi = 4\%$. It can be scrutinized that important growing of the Nusselt number with Reynolds number and volume friction of solid particles. The twist ratio 2 caused more disturbances in fluid flow at the core, therefore the Nusselt number improves. The twisted tape with twist ratio 2 increases the Nusselt number by $92.222\%$ compared to normal tube at $\phi = 1\%$ and $Re = 2000$. At $\phi = 2\%$ and $Re = 2000$, the twisted turbulator with twisted ratio 2 improves the estimations of Nusselt number by $90.662\%$ compared to without turbulator tube. The twisted tape with twisted ratio 2, enhance the Nusselt number by $90.522\%$ compared to normal tube at $\phi = 0.03$ and $Re = 2000$. For $\phi = 0.04$ and $Re = 2000$, the TT with twist ratio 2, improves the Nusselt number by $90.1005\%$ compared to without turbulator tube.

### Impact of pitch ratio on heat transfer coefficient

**Figure 6:**
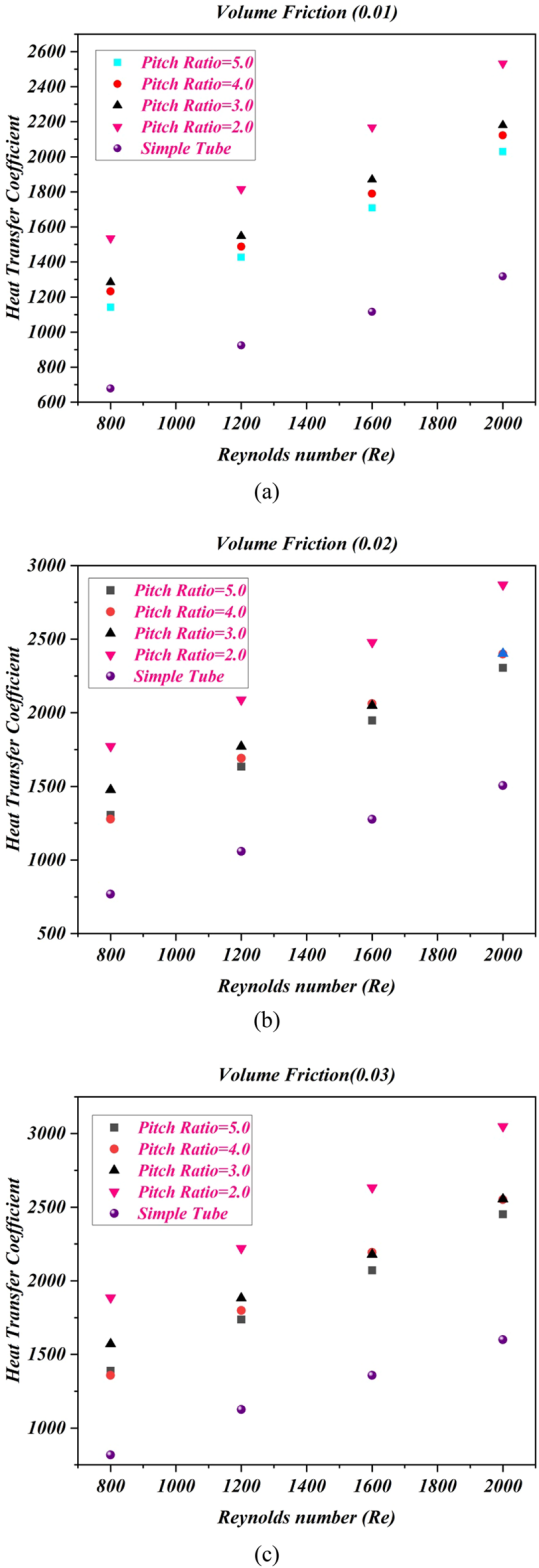

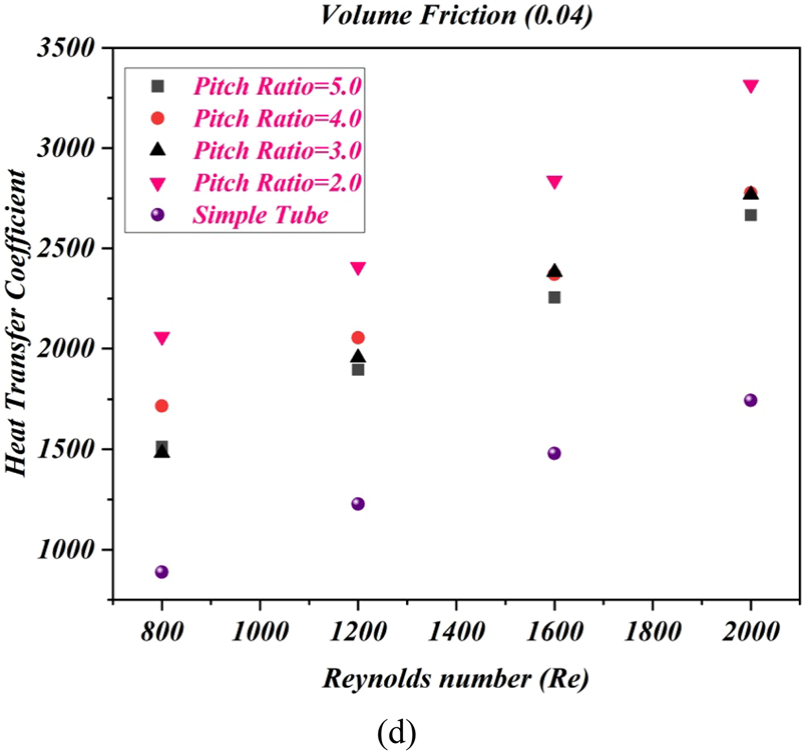
The estimations of heat transfer coefficient against $Re$ with captured different cases for (**a**) $\phi = 1\%$ (**b**) $\phi = 2\%$ (**c**) $\phi = 3\%$ (**d**) $\phi = 4\%$.

Figure 6 shows the heat transfer coefficient in terms of Reynolds number for cases (${p \mathord{\left/ {\vphantom {p D}} \right. \kern-0pt} D} = 2$,${p \mathord{\left/ {\vphantom {p D}} \right. \kern-0pt} D} = 2$, ${p \mathord{\left/ {\vphantom {p D}} \right. \kern-0pt} D} = 4$ and ${p \mathord{\left/ {\vphantom {p D}} \right. \kern-0pt} D} = 5$) at different variations of volume friction for all cases the heat transfer coefficient is an enhancing function of the Reynolds number and different volume friction of nanopowders. Here, it can be witnessed that the twisted tape with twist ratio 2, enhance the heat transfer coefficient as compared to simple tube and other cases. The fluctuation in the flow of fluid is more occurred due to twist pitch 44 mm at the core as compared to twist pitch 66 mm, 88 mm and 110 mm. Due to more fluctuation in fluid flow the heat transfer coefficient is increased for twist pitch 44 mm at different values of volume friction $\phi = 1\%$ to $\phi = 4\%$.

### Impact of pitch ratio on friction factor

**Figure 7:**
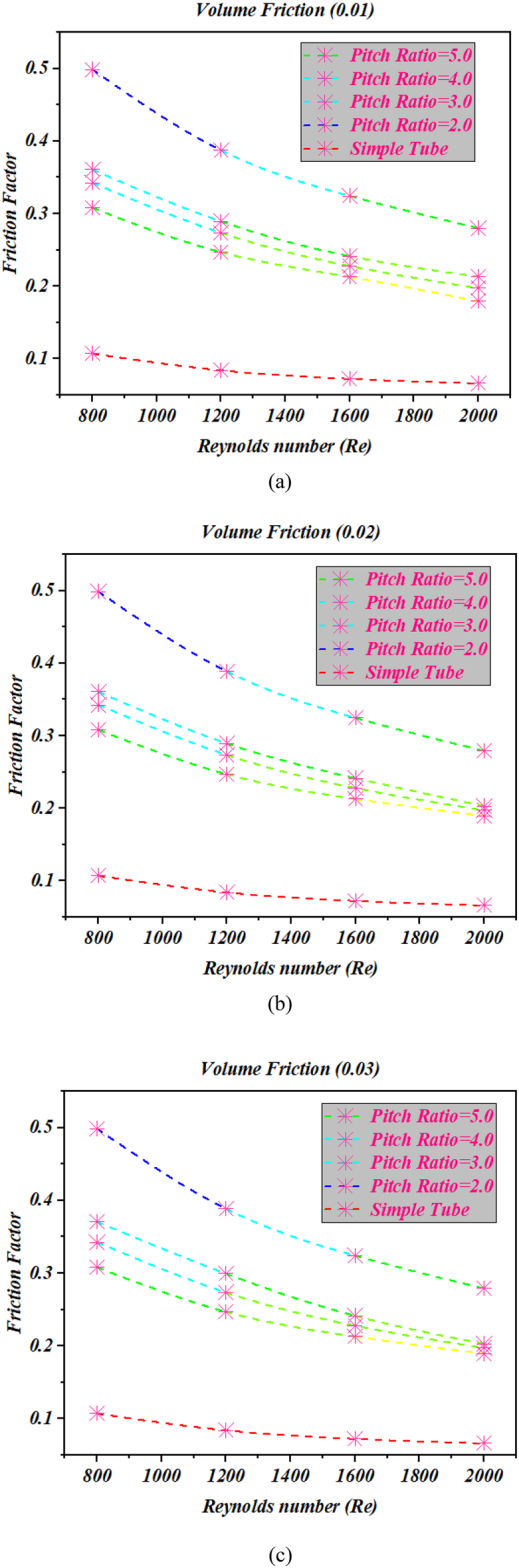

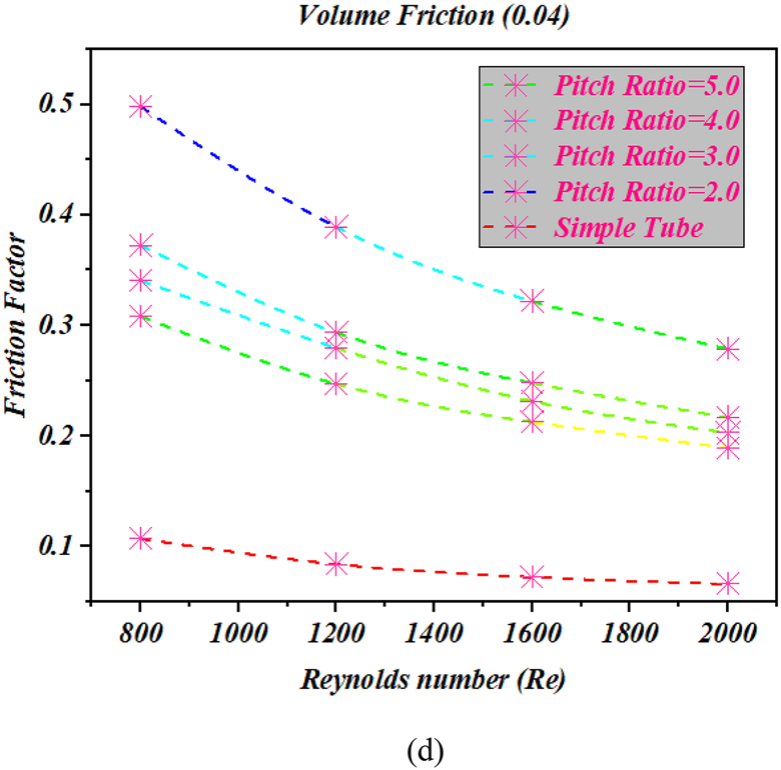
The estimations of friction factor against $Re$ with captured different cases for (**a**) $\phi = 1\%$ (**b**) $\phi = 2\%$ (**c**) $\phi = 3\%$ (**d**) $\phi = 4\%$.

**Figure 8:**
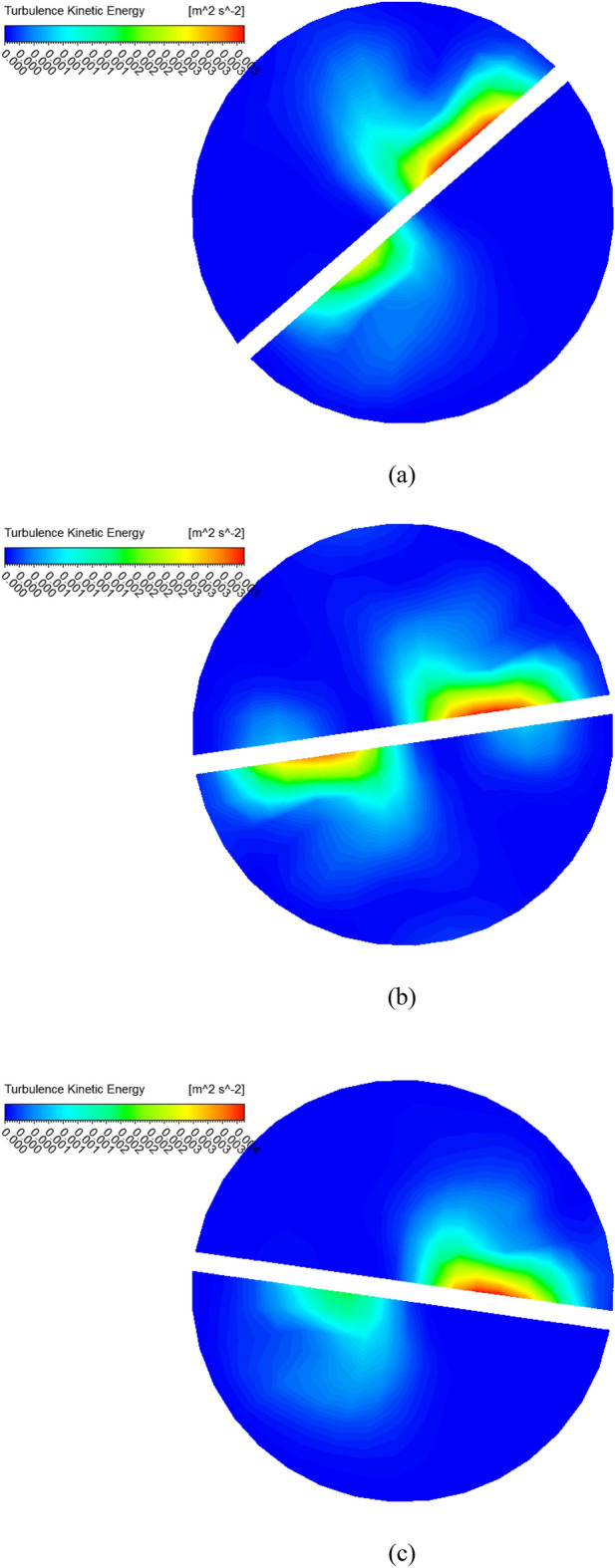
Turbulence kinetic energy contours.

In order to understand the impact of change in pitch of twisted tape in friction factor outcomes versus Reynolds number with different values of volume friction $1\%$ to $4\%$ and twist ratio 2,3,4 and 5 are considered as shown in Fig. 7 (a-d). The twist pitch 44 mm boosted up the friction factor against Reynolds number range 800, 1200, 1600, 200 at different volume friction of nanopowders $1\%$ to $4\%$. From the results it can be analyzed the twisted tape with twist pitch 44 mm and pitch ratio 2 boosted the friction factor as compared to twist pitch 66 mm, 88 mm and 110 mm. furthermore from the figure it can be witnessed that the friction factor is improves in all cases as compared to simple tube without twist tape (Fig. 8).

### Performance evaluation

To obtain the heat transfer evaluation the thermo-hydraulic performance factor us applied, which is addressed as:

17 $$\varepsilon = \frac{{Nu/Nu_{0} }}{{\left( {f_{r} /f_{0} } \right)^{1/3} }}$$

**Figure 9:**
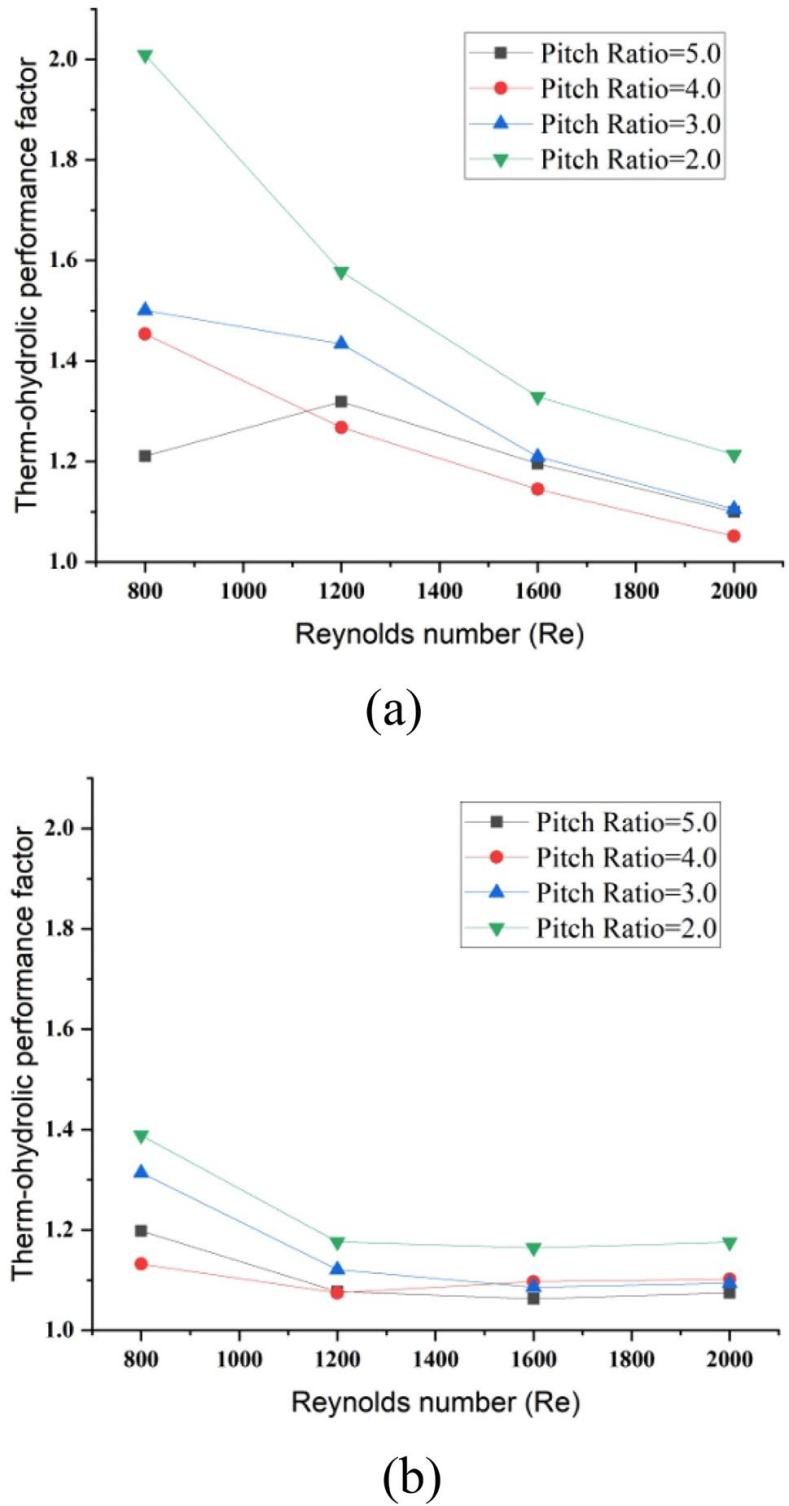
Thermal hydraulic performance for tube with twisted tape (**a**) water (**b**) hybrid nanofluid.

Here $Nu_{0}$ indicate the Nusselt number of the plain tube and $f_{o}$ be the friction factor of the plain tube. In Fig. 9a and b the thermo-hydraulic performance factor via different values of Reynolds number is elaborated for water and hybrid nanofluid.

## Conclusion

The significance of heat transfer and friction factor of incompressible hybrid nanofluid flow in tube with different pitch ratio of twisted tape was scrutinized numerically. The nanoparticles fraction range 1% to 4% is selected with range of Reynolds numbers of 800, 1200, 1600, 2000 and the pitch ratio of twisted tape is 2, 3, 4, and 5 is illustrated comprehensively. The CFD fluent is used to compute the simulation. Also the effects of Nusselt number, heat transfer coefficient and friction factor is computed in case of simple tube and twisted tape insert in tube. The major concluding remarks of current study are listed below:

- The heat transfer is boosted in case of declining the twist ratio and growing the values of Reynolds number.
- The heat transfer is more boosted up in case of twisted tape compared to without twisted tube.
- The pitch ratio 2 caused more disturbances in the flow of fluid, as a result Nusselt number increases for volume friction from $1\%$ to $4\%$ and the value of Reynolds number is 2000.
- The coefficient of heat transfer is boosted up for twist tape with pitch ratio 2 insert in tube at different values of volume friction.
- Also, friction factor is increases via increases Reynolds number at pitch ratio of twisted tape is 2 and volume friction of nanoparticles is $1\%$ to $4\%$.
- Because of an increase in axial convection, the heat transfer improves as the Reynolds number rises. Because of the disturbed boundary layer caused by a rise in Reynolds number, the heat transmission from the wall to the fluid is amplified.
- Higher Reynolds number improves in hybrid nanofluid volume friction from $1\%$ to $4\%$ enhance the Nusselt number and friction factor by $8.45\%$ and $3.35\%$ respectively.

The choice of a twisted tape in current article is justified as it enhances turbulence, promotes effective heat transfer, and is a well-established method for improving heat transfer efficiency. It offers practicality in implementation, ease of parameter variation, and a proven track record in various applications, making it a suitable and effective choice for this study aiming to predict the potential of hybrid nanofluids.

## Data Availability

The datasets used and/or analyzed during the current study available from the corresponding author (D. L.) on reasonable request.
